# A Primary Health Care Program and COVID-19. Impact in Hospital Admissions and Mortality

**DOI:** 10.1007/s11606-024-08912-6

**Published:** 2024-07-18

**Authors:** Cristina García-Marichal, Manuel Francisco Aguilar-Jerez, Luciano Jonathan Delgado-Plasencia, Onán Pérez-Hernández, José Fernando Armas-González, Ricardo Pelazas-González, Candelaria Martín-González

**Affiliations:** 1 Gerencia de Atención Primaria de Tenerife, San Cristóbal De La Laguna, Spain; 2 Gerencia de Atención Primaria de Tenerife, Arico, Spain; 3 Hospital Universitario de Canarias, San Cristóbal De La Laguna, Spain; 4 Universidad de La Laguna, San Cristóbal De La Laguna, Spain; 5 Departamento de Medicina Interna, Dermatología y Psiquiatría, Universidad de La Laguna, Servicio de Medicina Interna, Hospital Universitario de Canarias, Canary Islands, Tenerife, Spain

**Keywords:** primary health care program, COVID-19, vulnerability factors, telemedicine, mortality

## Abstract

**Background:**

Most patients with mild or moderate COVID infection did not require hospital admission, but depending on their personal history, they needed medical supervision. In monitoring these patients in primary care, the design of specific surveillance programs was of great help. Between February 2021 and March 2022, EDCO program was designed in Tenerife, Spain, to telemonitor patients with COVID infection who had at least one vulnerability factor to reduce hospital admissions and mortality.

**Objective:**

The aim of this study is to describe the clinical course of patients included in the EDCO program and to analyze which factors were associated with a higher probability of hospital admission and mortality.

**Design:**

Retrospective cohort study.

**Patients:**

We included 3848 patients with a COVID-19 infection age over 60 years old or age over 18 years and at least one vulnerability factor previously reported in medical history.

**Main Measures:**

Primary outcome was to assess risk of admission or mortality.

**Key Results:**

278 (7.2%) patients required hospital admission. Relative risks (RR) of hospital admission were oxygen saturation ≤ 92% (RR: 90.91 (58.82–142.86)), respiratory rate ≥ 22 breaths per minute (RR: 20.41 (1.19–34.48), obesity (RR: 1.53 (1.12–2.10), chronic kidney disease (RR:2.31 (1.23–4.35), ≥ 60 years of age (RR: 1.44 (1.04–1.99). Mortality rate was 0.7% (27 patients). Relative risks of mortality were respiratory rate ≥ 22 breaths per minute (RR: 24.85 (11.15–55.38), patients with three or more vulnerability factors (RR: 4.10 (1.62–10.38), oxygen saturation ≤ 92% (RR: 4.69 (1.70–15.15), chronic respiratory disease (RR: 3.32 (1.43–7.69) and active malignancy (RR: 4.00 (1.42–11.23).

**Conclusions:**

Vulnerable patients followed by a primary care programme had admission rates of 7.2% and mortality rates of 0.7%. Supervision of vulnerable patients by a Primary Care team was effective in the follow-up of these patients with complete resolution of symptoms in 91.7% of the cases.

## INTRODUCTION

On 31 December 2019, the Chinese government first reported an outbreak of Coronavirus disease (Covid-19) in Wuhan, the capital of China's Hubei province. The pandemic spread rapidly throughout China's provinces and to the rest of the world.^1^

Since the beginning of the pandemic, attempts have been made to identify poor prognostic risk factors in patients with Covid-19 infection^2^ that lead to hospital admission or increased risk of death.^3–5^ Many of the risk factors identified in patients with COVID infection with a worse clinical course overlap with the so-called vulnerability factors. In 2021, the Spanish Ministry of Health defined a set of criteria identifying patients as vulnerable to infection and therefore more susceptible to contracting the disease and having a worse clinical course once infected.^6,7^

Primary Care is the first level of care, and, therefore, should play a crucial role in the response to the pandemic. The COVID-19 pandemic led to the reorganization of Primary Care to cover the situation, implementing the double care circuit, with differentiated areas to separate care for patients with symptoms compatible with COVID-19 from the rest, together with the reduction of face-to-face activity to avoid contagion, the increase in telephone medical consultations and the use of virtual consultation systems between professionals. Teams of healthcare professionals were also structured to support various health centers to facilitate access to COVID-19 diagnostic tests and the vaccination campaign.^8^ On the other hand, in recent years and because of technological development, telemedicine or non-face-to-face patient care is being implemented in different countries.^9,10^ COVID-19 pandemic conditioned a rapid implementation of some telemedicine programs since, given the situation of overloaded care and the need to provide medical care to an increasing number of patients, it was proposed that Primary Care systems should design strategies to identify which patients were candidates to earlier care and stricter supervision.^4,5,11^

Most patients with mild or moderate COVID infection did not require hospital admission, but depending on their personal history, they needed medical supervision. In monitoring these patients in primary care, the design of specific surveillance programs was of great help. Thus, several programs were designed in different countries^12–14^ and included one or various visits per patient, performed by physicians or nurses, who assessed symptoms and clinical status (oxygen saturation, blood pressure or heart rate, among others). Each protocol established criteria for hospital referral either for emergency department assessment or hospital admission.^15^ In our area, the EDCO program (Covid home care team) was designed, which serves a reference population of approximately 950000 inhabitants. Inclusion criteria were patients with a COVID-19 infection confirmed by real-time reverse transcription-polymerase chain reaction (RT-PCR), age over 60 years old or age over 18 years and at least one vulnerability factor previously reported in medical history. At inclusion, patients were evaluated by a medical doctor at home, and data collected included demographics, clinical characteristics, and clinical symptoms. On subsequent days patients were called once a day or video-called depending on their clinical status.

The main objective of this study is to describe the clinical course of patients included in the EDCO program and to analyze which factors were associated with a higher probability of hospital admission and mortality.

## MATERIAL AND METHODS

A total of 3848 patients (2145 -55.7%- women) were retrospectively included, with a mean age of 57.5 ± 17.2 years, consecutively recruited in a Primary Care project designed to closely monitor vulnerable patients with the aim of reducing morbidity and mortality. Patients were recruited between 1/02/2021 to 30/03/2022. Inclusion criteria were patients with a COVID-19 infection confirmed by real-time reverse transcription-polymerase chain reaction (RT-PCR), age over 60 years old or age over 18 years and at least one vulnerability factor previously reported in medical history: diabetes mellitus type-1 and type-2, hypertension, severe neurological disease (Alzheimer's disease, epilepsy, stroke, Parkinson's disease, brain tumors, multiple sclerosis), chronic heart disease (including chronic ischemic heart disease, heart failure, cardiomyopathy), chronic respiratory disease (including chronic obstructive pulmonary disease -COPD-, asthma, pulmonary fibrosis, sleep apnoea-hypopnoea syndrome, chronic bronchitis, pulmonary emphysema, interstitial diseases and bronchiectasis), home oxygen therapy, active malignancy, chronic kidney disease (including haemodialysis and kidney transplantation), chronic liver disease (alcoholic liver disease or non-alcoholic steatohepatitis or autoimmune liver disease), obesity (body mass index > 30 kg/m^2^), immunosuppression and pregnancy.

Before patients were included in the EDCO program, nursing and medical staff were trained. Training was provided to acquire skills in the use of personal protective equipment and staff were trained to perform physical examinations and recognize warning signs for early referral to the hospital. Continuous updates on Spanish Ministry of Health protocols were also provided through online clinical sessions and staff were instructed in the use of video call as a method of communication.

As mentioned above, at inclusion, patients were evaluated by a medical doctor at home. Home visits were conducted in a private vehicle. On the first visit, data collected included demographics, clinical characteristics such as basal oxygen saturation, blood pressure, temperature, heart rate and respiratory rate, among others and clinical symptoms (Table 1). Medical doctor transmitted data collected by telephone to the staff present at the health center, and these were compiled in the patient’s medical record. At the discretion of the physician, patients who needed it were given a pulse oximeter. Patients and/or their relatives were instructed to take at least once a day or if clinical changes appeared several vital signs such as: respiratory rate, heart rate, temperature, blood pressure and oxygen saturation if a pulse oximeter was available, and, also we trained them to identify alarm symptoms (hypotension, decreased oxygen saturation, altered consciousness, tachypnoea, respiratory distress, or prolonged fever). If they worsened, they should request referral to the emergency department. Patients were followed up by telephone by their primary care physician daily and vital signs were requested from the patients or their relatives. This data was recorded daily in the medical record. Patients with worsening symptomatology or who showed any alarm signs during the surveillance period were assessed by the physician by video call. Criteria for referral to hospital were quick SOFA score ≥ 2 (qSOFA score includes arterial hypotension (systolic blood pressure ≤ 100 mmHg), impaired level of consciousness or tachypnoea (respiratory rate ≥ 22 respirations per minute)),^16^ temperature ≥ 38ºC for three days in a row, basal oxygen saturation ≤ 92%. In addition to the established referral criteria, the physician could decide to refer a patient to hospital if he/she considered it appropriate. Patients who required hospitalization underwent blood analysis and chest radiography.

**Table 1:** Demographic Variables, Comorbidities, Symptoms, and Vital Signs of Patients with Covid-19

Characteristics	n	%	Mean ± Standard Deviation Mediana (Interquartile range)
Sex			
Male	1703	44,3	
Female	2145	55,7	
Age in years			
< 60	1871	48,6	
≥ 60	1977	51,4	
Comorbidity			
Diabetes Mellitus	976	25,4	
Hypertension	2191	56,9	
Severe neurological disease	168	4,4	
Chronic heart disease	817	21,2	
Chronic respiratory disease	1102	28,6	
Home oxygen therapy	6	0,2	
Active malignancy	181	4,7	
Chronic kidney disease	136	3,5	
Chronic liver disease	89	2,3	
Obesity	1333	34,6	
Immunosuppression	129	3,4	
Pregnancy	94	2,4	
Vulnerability factors^1^			
≥ 2 factors	2609	67,8	
≥ 3 factors	1525	39,6	
Immunization			
No record	2	0,1	
Not vaccinated	1871	48,6	
Incomplete vaccination	486	12,6	
Complete vaccination	1487	38,6	
Symptoms			
Cough	1578	41	
Expectoration	544	14,1	
Pleuritic pain	98	2,5	
Diarrhea	274	7,1	
Vomiting	58	1,5	
Dyspnea	459	11,9	
Vital Signs			
Temperature in ºC	3845		36.3 ± 0.53 36.2 (36–36.5)
Oxygen Saturation	3837		97.2 ± 2.64 98 (97–98)
Systolic blood pressure	3803		131.56 ± 18.1 131 (120–142)
Diastolic blood pressure	3803		80.6 ± 11.42 80 (72–89)
Heart rate	3834		78.45 ± 13.32 78 (69–87)
Respiratory rate	3839		15.8 ± 2.3 15 (14–18)

^1^Vulnerability factors: age over 60 years, diabetes mellitus type 1 and type 2, hypertension, severe neurological disease, chronic heart disease, chronic respiratory disease, home oxygen therapy, active malignancy, chronic kidney disease, chronic liver disease, obesity, immunosuppression, and pregnancy

## OUTCOMES

The primary outcome was hospital admission. Secondary outcomes were admission to the critical care unit and mortality.

## ETHICS

The study protocol was approved by the local ethical committee of our Hospital (CHUC_2022_72) and was conducted in accordance with the ethical guidelines of the 1975 Declaration of Helsinki (revised in 2013). To comply with the privacy policy, all patient identification data were removed. Written consent was not collected during pandemic peak to avoid paper contamination, in accordance with sanitary dispositions at that time and, the research ethics committee granted an exemption from informed consent.

## STATISTICAL ANALYSIS

The Kolmogorov–Smirnov test was used to explore if the variables showed a normal distribution or not. Demographic and clinical characteristics of patients were presented as mean ± standard deviation (SD) or percentages for categorical variables. For continuous variables that did not follow a normal distribution, data were reported as median and interquartile range (IQR). Mann-Whithney´s U (Z) test was used when the variables included in any of these analyses showed a non-parametric distribution, whereas Student's t test was used with the variables with a normal distribution. χ2 test was used to compare qualitative variables. Survival was analyzed using Kaplan Meier curves. We also performed a multivariate analysis with those parameters that were significantly related to hospital admission or mortality in univariate analysis, to test the independence or not of the relationships between hospital admission and several risk/vulnerability factors and between mortality and several risk/vulnerability factors. Mortality analysis includes variables obtained at the time of hospital admission, such as analytical variables. Analyses were performed with SPSS software (25.0) (Chicago, Ill., USA).

## RESULTS

A total of 3848 patients (55.7% female) were included. The median age was 60 (46–70) years, with 51.4% (1977) were ≥ 60 years of age. Vulnerability factors, in addition to age, were hypertension (56.9%), obesity (34.6%), chronic respiratory disease (28.6%), diabetes mellitus (25.4%), chronic heart disease (21.2%), active neoplasia (4.7%), severe neurological disease (4.4%), renal disease (3.5%), immunosuppression (3.4%), pregnancy (2.4%), home oxygen therapy (0.2%) (Table 1). Regarding the prevalence of vulnerability factors, 2609 (67.8%) patients had 2 or more vulnerability factors, while 1525 (39.6%) patients had 3 or more vulnerability factors.

Clinical characteristics are shown in Table 1. The most frequent symptoms were cough (41%) followed by expectoration (14.2%) and dyspnea (11.9%). The average symptom duration was 4.2 ± 1.3 days. 3527 patients (91.7%) experienced mild to moderate illness with resolution of symptoms at the time of program discharge. 1871 (48.2%) patients were not vaccinated at the time of the study, while 486 (12.6%) had received 1 dose of vaccine and 1487 (38.6%) of them 2 doses of vaccine. Vaccination data were not available for 2 patients.

Only 321 (8.3% of total) patients required emergency care, of whom 278 (7.2% of total) were admitted to the hospital and 57 (1.5%) patients required admission to the critical care unit. 105 (37.8%) of the admitted patients had pneumonia on chest X-ray at the time of admission. Univariate analysis revealed multiple risk factors for admission (Table 2). Admitted patients were older (T = 7.70; p < 0.001) and mainly men (χ^2^ = 4.89; p = 0.027). Most vulnerability factors were related to admission: diabetes mellitus (31% Vs 25%; χ^2^ = 4.03; p = 0.045), severe neurological disease (8.3% Vs 4.1%; χ^2^ = 9.92; p = 0.002), chronic heart disease (26.3% Vs 20.1%; χ^2^ = 4.25; p = 0.039), renal disease (7.9% Vs 3.2%; χ^2^ = 15.74; p < 0.001), obesity (44.2% Vs 33.8%; χ^2^ = 11.89; p = 0.001). Patients with two or more (χ^2^ = 20.70; p < 0.001) or three or more (χ^2^ = 23.96; p < 0.001) vulnerability factors needed admission more frequently. Systolic blood pressure ≤ 100 mmHg and respiratory rate ≥ 22 breaths per minute at home assessment were associated with increased risk of hospital admission (p ≤ 0.001 in both cases). Some biochemical parameters were significantly higher in patients who required hospitalization, such as serum creatinine (Z = 1.97; p = 0.049), ferritin (Z = 2.87; p = 0.004), lactate dehydrogenase (Z = 2.84; p = 0.005); aspartate aminotransferase (Z = 3.29; p = 0.001), alanine aminotransferase (Z = 2.76; p = 0.006), C-reactive protein (Z = 2.30; p = 0.021) and procalcitonin (Z = 3.75; p ≤ 0.001) (Table 2).

**Table 2:** Risk Factors for Admission and Mortality

	**Hospital admission (n = 278)**			**Mortality (n = 27)**		
Sex^**1**^	Male 50.7% (N = 141)	Women 49.3% (N = 137)	X2 = 4.89; p = 0.027	Male 48.1% (N = 13)	Women 51.9% (N = 14)	X2 = 0.006; NS
Age^**1**^	≥ 60 years 65.1% (N = 181)	< 60 years 34.9% (N = 97)	X2 = 22.19; p < 0.001	≥ 60 years 74.1% (N = 20)	< 60 years 25.9% (N = 7)	X2 = 4.73; p = 0.030
**Comorbility**	**Hospital admission (N = 278)**	**Not admitted to hospital (N = 3570)**	**p**	**Dead (N = 27)**	**Alive (N = 3821)**	**p**
Diabetes Mellitus^**1**^	31.0% (N = 85)	25.0% (N = 889)	X2 = 4.03; p = 0.045	44.4% (N = 12)	25.2% (N = 964)	X2 = 4.26; p = 0.039
Hypertension^**1**^	62.6% (N = 174)	56.5% (N = 2014)	X2 = 3.65; p = 0.056	63.0% (N = 17)	56.9% (N = 2174)	X2 = 0.19; NS
Severe neurological disease^**1**^	8.3% (N = 23)	4.1% (N = 145)	X2 = 9.92; p = 0.002	11.1% (N = 3)	4.3% (N = 165)	X2 = 1.56; NS
Chronic heart disease^**1**^	26.3% (N = 73)	20.8% (N = 742)	X2 = 4.25; p = 0.039	37.0% (N = 10)	21.1% (N = 807)	X2 = 3.17; p = 0.075
Chronic respiratory disease^**1**^	29.5% (N = 82)	28.5% (N = 1016)	X2 = 0.08, NS	48.1% (N = 13)	28.5% (N = 1089)	X2 = 4.15; p = 0.042
Active malignancy^**1**^	7.2% (N = 20)	4.5% (N = 160)	X2 = 3.65, P = 0.056	22.2% (N = 6)	4.6% (N = 175)	X2 = 14.89; p < 0.001
Chronic kidney disease^**1**^	7.9% (N = 22)	3.2% (N = 113)	X2 = 15.74; p < 0.001	11.1% (N = 3)	34.82% (N = 133)	X2 = 2.61; NS
Chronic liver disease^**1**^	1.4% (N = 4)	2.4% (N = 84)	X2 = 0.60, NS	7.4% (N = 2)	2.3% (N = 87)	X2 = 1.27; NS
Obesity^**1**^	44.2% (N = 123)	33.8% (N = 1206)	X2 = 11.89; p = 0.001	29.6% (N = 8)	34.7% (N = 1325)	X2 = 0.12; NS
Immunosuppression^**1**^	2.9% (N = 8)	3.3% (N = 119)	X2 = 0.06; NS	14.8% (N = 4)	3.3% (N = 125)	X2 = 7.75; p = 0.005
Pregnancy^**1**^	1.1% (N = 3)	2.6% (N = 91)	X2 = 1.77; NS	0.0% (N = 0)	2.5% (N = 94)	X2 = 0.04; NS
≥ 2 factors of vulnerability^**1**^	80.2% (N = 223)	66.8% (N = 2380)	X2 = 20,70; p < 0.001	96.3% (N = 26)	67.6% (N = 2583)	X2 = 8.84; p = 0.003
≥ 3 factors of vulnerability^**1**^	53.6% (N = 149)	38.5% (N = 1372)	X2 = 24.00; p < 0.001	74.1% (N = 20)	39.4% (N = 1505)	X2 = 12.07; p = 0.001
Systolic blood pressure ≤ 100 mmHg^**2**^	9.9% (N = 27)	4.4% (N = 155)	X2 = 15.40; p < 0.001	18.5% (N = 5)	4.7% (N = 177)	X2 = 8.42; p = 0.004
Respiratory rate ≥ 22 breaths per minute^**2**^	30.7% (N = 84)	1.0% (N = 35)	X2 = 734.84; p < 0.001	40.7% (N = 11)	2.9% (N = 109)	X2 = 114.85; p < 0.001
Oxygen saturation ≤ 92%^**2**^	44.1% (N = 120)	0.8% (N = 30)	X2 = 1246.35; p < 0.001	42.3% (N = 11)	3.6% (N = 139)	X2 = 92.71; p < 0.001
qSOFA Score ≥ 2^**2**^	3.3% (N = 9)	0.3% (N = 10)	X2 = 40.67; p < 0.001	11.1% (N = 3)	0.4% (N = 16)	X2 = 42.44; p < 0.001
Pneumonia^**3**^	37.8% (N = 105)	1.1% (N = 1)	X2 = 42.78; p < 0.001	96% (N = 24)	32.9% (N = 81)	X2 = 35.43; p < 0.001
**Biochemical parameters**^**3**^						
pO2 in blood gases	52.86 ± 20.77 55 (38–67)	48.22 ± 21.23 47 (29–71)	Z = 0.53; NS	46.9 ± 13.8 47 (37.5–59)	53.33 ± 21.15 56.5 (36.5–67.8)	Z = 1.54; NS
Lymphocytes (x10e^6^/L)	1397 ± 4844 950 (640–1380)	1216 ± 712 1205 (795–1657)	Z = 1.10; NS	850 ± 617 640 (432–1123)	1400 ± 4996 960 (670–1380)	Z = 2.50; p = 0.012
Leukocytes (x10e^6^/L)	6787 ± 6781 5950 (4095–7805)	6073 ± 4006 5885 (3602–7495)	Z = 0.43; NS	5961 ± 3673 4985 (3362–9363)	6873 ± 7018 6100 (4280–7800)	Z = 1.15; NS
Neutrophils (x10e^6^/L)	5822 ± 9119 4360 (2605–6305)	4452 ± 3187 4185 (2137–5870)	Z = 0.47; NS	8045 ± 16,175 4120 (2407–7042)	5601 ± 8098 4380 (2610–6290)	Z = 0.03; NS
Platelets (x10e^6^/L)	215 ± 100 192 (151–253)	277 ± 131 239 (147–428)	Z = 1.33; NS	171 ± 90,9 155 (113–241)	221 ± 101 195 (153–255)	Z = 2.40; p = 0.016
Creatinine mg/dL	1.05 ± 0.78 0.91 (0.76–1.11)	0.80 ± 0.17 0.74 (0.66–0.96)	Z = 1.97; p = 0.049	1.39 ± 1.39 1.02 (0.68–1.57)	1.02 ± 0.69 0.89 (0.76–1.09)	Z = 1.12; NS
Ferritin (ng/mL)	998 ± 1494 641 (297–1259)	328 ± 440 190 (116–273)	Z = 2.87; p = 0.004	1969 ± 3953 768 (263–1786)	884.23 ± 846 623 (289–1214)	Z = 1.01; NS
Lactate Dehydrogenase (U/L)	344 ± 116 315 (262–418)	233 ± 69.3 217 (182–291)	Z = 2.84; p = 0.005	413 ± 130 407 (304–494)	335, ± 113 308 (253–403)	Z = 2.89; p = 0.004
Aspartate aminotransferase (U/L)	47.8 ± 38 38 (27–57)	23.6 ± 7,2 25 (17.2–30.5)	Z = 3.29; p = 0.001	47.9 ± 31.08 36 (27–68)	47.42 ± 38.7 38 (27–56.3)	Z = 0.32; NS
Alanine aminotransferase (U/L)	47.9 ± 46.3 34 (22–58)	21.5 ± 8.1 23.5 (15.3–26)	Z = 2.76; p = 0.006	37.25 ± 33.65 25 (17.5–48.5)	48.64 ± 47.1 34 (23–59)	Z = 1.69; p = 0.091
D-dimer (ng/ml)	3642 ± 15,309 729 (468–1157)	854 ± 1183 516 (229–814)	Z = 1.84; p = 0.066	5451 ± 20,027 787 (553–1505.5)	3440 ± 14,708 716 (466–1151)	Z = 0.38; NS
C-Reactive Protein (mg/dL)	45 ± 64.5 17 (7–60)	20.02 ± 32.2 4.47 (0.78–34.3)	Z = 2.30; p = 0.021	64.9 ± 66.4 48 (14.6–85.7)	42.8 ± 63.81 14.54 (6.52–51.96)	Z = 2.86; p = 0.004
Procalcitonin (ng/ml)	0.62 ± 4.6 0.09 (0.05–0.18)	0.03 ± 0.02 0.02 (0.02–0.05)	Z = 3.75; p < 0.001	1.39 ± 5.12 0.23 (0.09–0.44)	0.54 ± 4.49 0.08 (0.05–0.16)	Z = 3.58; p < 0.001
Fibrinogen (mg(dL)	714 ± 176 714 (601–841)	567.2 ± 173 500 (428–739)	Z = 1.90; p = 0.057	719.2 ± 123.64 762 (587–800)	713.2 ± 181.35 701 (601.8–847.3)	Z = 0.11; NS
Interleukin 6 (ng/L)	57.7 ± 87.6 29.9 (15.2–63.7)	27.45 ± 33.3 27.45 (3.9–27.45)	Z = 0.59; NS	162.7 ± 199 78.4 (28.2–234.5)	47.4 ± 59.7 28.9 (14–59.2)	Z = 2.82; p = 0.005

^1^Measured at enrolment into study, ^2^measured at home later, ^3^measured at admission

On multivariate analysis, the relative risks (RR) of hospital admission were respiratory rate ≥ 22 (RR: 20.41 (1.19–34.48), obesity (RR: 1.53 (1.12–2.10), chronic kidney disease (RR:2.31 (1.23–4.35), ≥ 60 years of age (RR: 1.44 (1.04–1.99) (Table 3).

**Table 3:** Risk Ratios (95% confidence interval) of Hospital Admission (multivariate analysis)

Factor	RR	95% CI
Respiratory rate ≥ 22 breaths per minute^1^	20.41	1.19–34.48
Obesity (BMI > 30 kg/m^2^)^2^	1.53	1.12–2.10
Chronic kidney disease^2^	2.31	1.23–4.35
≥ 60 years of age^2^	1.44	1.04–1.99

^1^Measured at home after enrolment into study, ^2^measured at enrolment into study

The overall mortality rate was 0.7% (27 patients). All patients died in hospital. Risks factors for mortality were age (68.78 ± 12.26 Vs 57.43 ± 17.21; T = 26.73; p < 0.001), diabetes mellitus (44.4% Vs 25.2%; χ^2^ = 4.26; p = 0.039), chronic respiratory disease (48.1% Vs 28.5%; χ^2^ = 4.15; p = 0.042), active malignancy (22.2% Vs 4.6%; χ^2^ = 14.89; p < 0.001), immunosuppression (14.8% Vs 3.3%, χ^2^ = 7.75; p = 0.005). Patients with two or more (χ^2^ = 8.84; p = 0.003) or three or more (χ^2^ = 12.07; p = 0.001) vulnerability factors had higher risk of mortality. Also, patients with clinical signs of severity such us systolic blood pressure ≤ 100 mmHg, respiratory rate ≥ 22 breaths per minute, oxygen saturation ≤ 92% or qSOFA Score ≥ 2 at home assessment were associated with increased mortality (p ≤ 0.005 in all cases). In relation to laboratory markers, lymphopenia (Z = 2.50; p = 0.012), thrombopenia (Z = 2.40; p = 0.016) and higher levels of serum LDH (Z = 2.89; p = 0.004), C-reactive protein (Z = 2.86; p = 0.004), procalcitonin (Z = 3.58; p < 0.001) and IL-6 (Z = 2.82; p = 0.005) were associated with mortality (Table 2).

**Figure 1:**
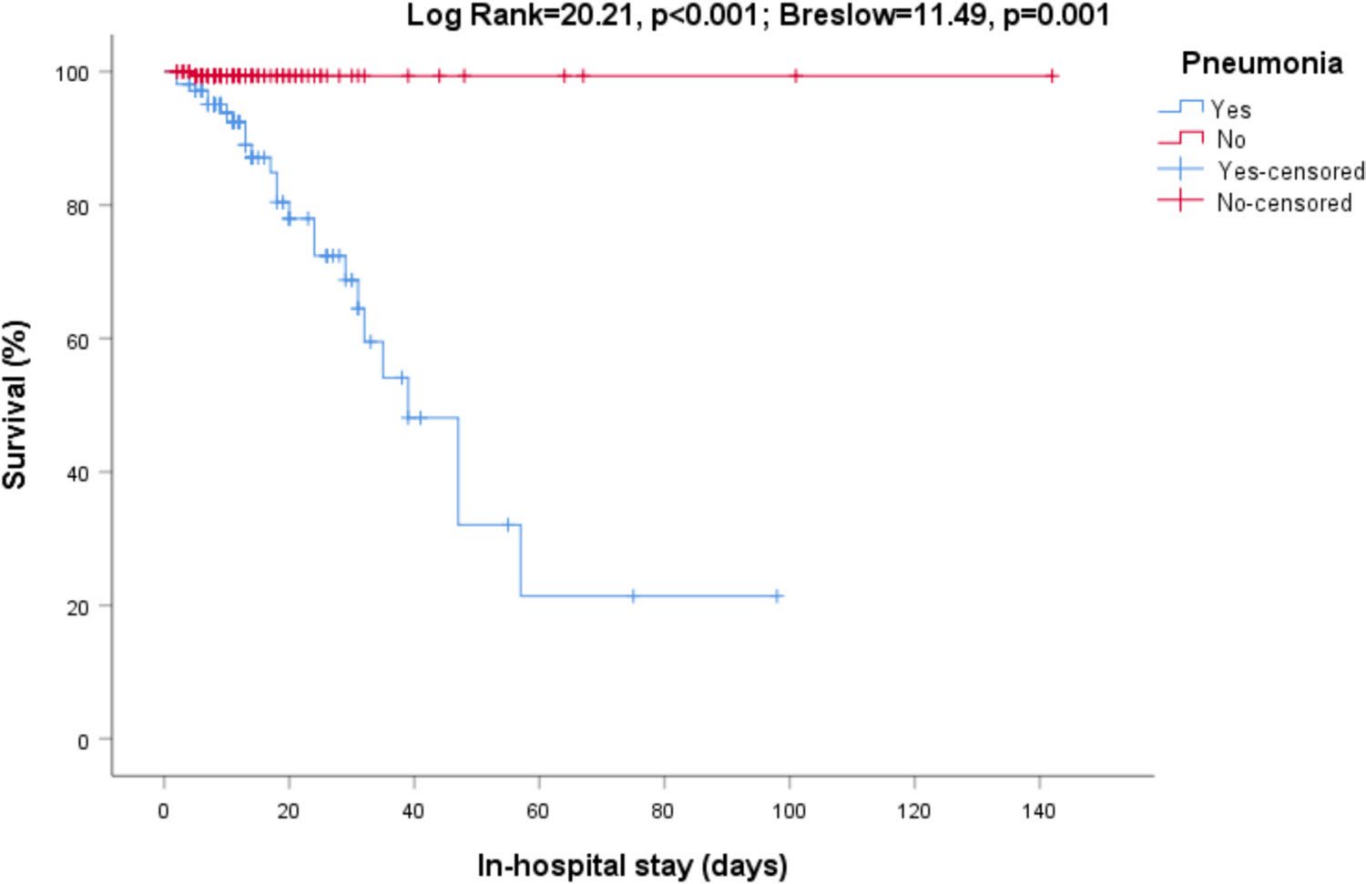
In-hospital mortality. Relationship between survival and pneumonia at admission (Log Rank = 20.21, p < 0.001; Breslow = 11.49, p = 0.001).

**Figure 2:**
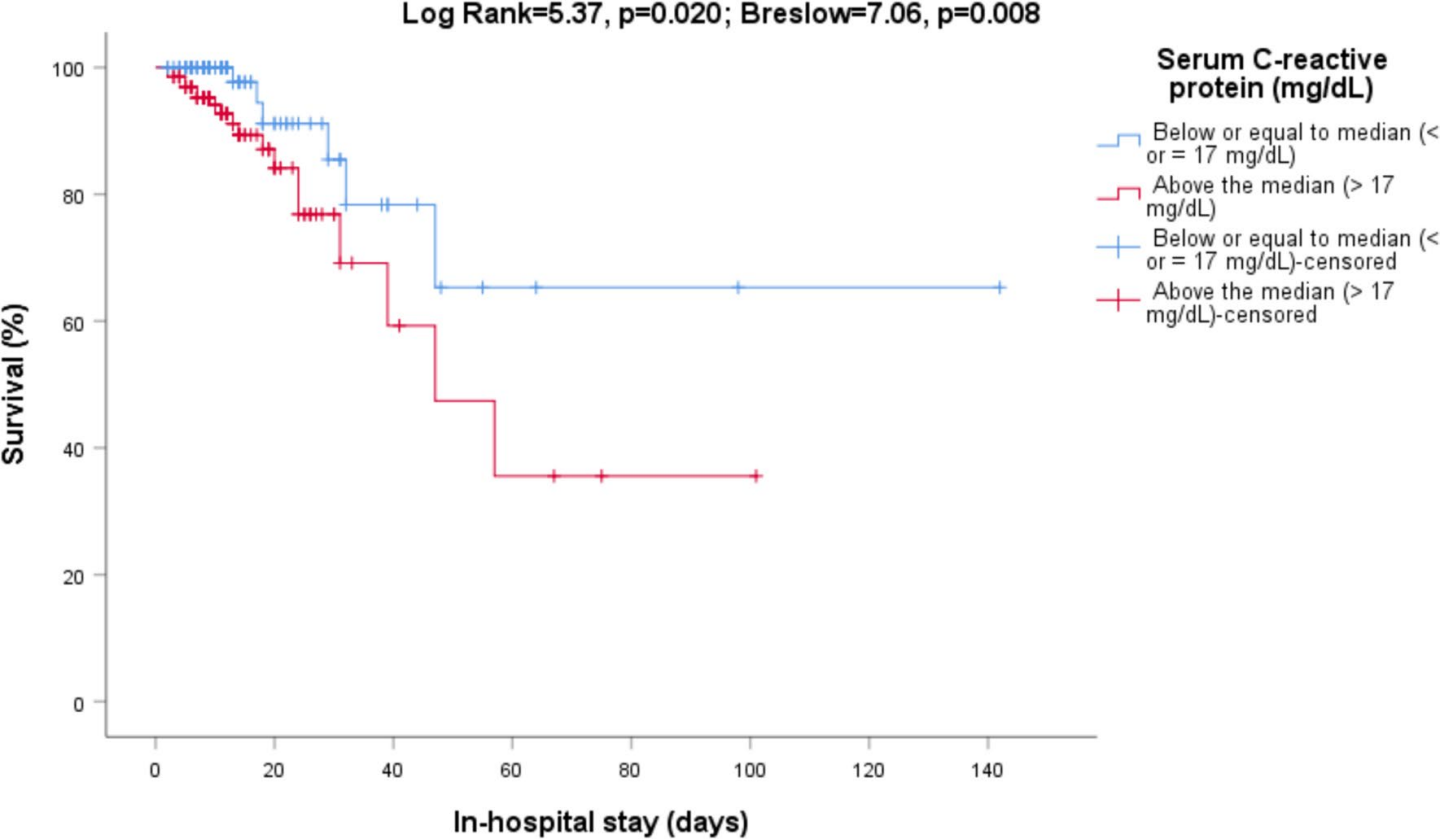
In-hospital mortality. Relationship between survival and serum C-reactive protein levels (Log Rank = 5.37, p = 0.020; Breslow = 7.06, p = 0.008).

In the univariate analysis performed with the Kaplan–Meier method, we found higher mortality among patients with pneumonia (Log Rank = 20.21, p < 0.001; Breslow = 11.49, p = 0.001, Fig. 1), active malignancy (Log Rank = 8.89, p = 0.003; Breslow = 7.47, p = 0.006) and chronic liver disease (Log Rank = 26.29, p < 0.001; Breslow = 17.37, p < 0.001). Also, values above the median of serum C-reactive protein (Log Rank = 5.37, p = 0.020; Breslow = 7.06, p = 0.008, Fig. 2) were associated with higher in-hospital mortality.

On multivariate analysis, the relative risks (RR) of mortality were respiratory rate ≥ 22 breaths per minute (RR: 24.85 (11.15–55.38), patients with three or more vulnerability factors (RR: 4.10 (1.62–10.38), oxygen saturation ≤ 92% (RR: 4.69 (1.70–15.15), chronic respiratory disease (RR: 3.32 (1.43–7.69) and active malignancy (RR: 4.00 (1.42–11.23) (Table 4).

**Table 4:** Risk Ratios (95% confidence interval) of Mortality (multivariate analysis)

Factor	RR	95% CI
Respiratory rate ≥ 22 breaths per minute^1^	24.85	11.15–55.38
≥ 3 factors of vulnerability^2^	4.10	1.62–10.38
Oxygen saturation ≤ 92%^1^	4.69	1.70–15.15
Chronic respiratory disease^2^	3.32	1.43–7.69
Active malignancy^2^	4.00	1.42–11.23

^1^Measured at home after enrolment into study, ^2^measured at enrolment into study

## DISCUSSION

The present retrospective cohort study describes the clinical and epidemiological characteristics of 3848 patients with mild-moderate Covid 19 infection with at least one vulnerability factor included in a Primary Care remote monitoring program. It was one of the first outpatient monitoring programs developed in Spain. There are few published studies involving remote monitoring programs for COVID patients, and those that do include substantially fewer patients, although experience with home monitoring programs is growing,^17–19^ including post-discharge monitoring of patients with covid infection who required hospital admission.^20^

In our series the mean age was 60 years, and all patients had some underlying pathology. Pimlott et al. published the results of a virtual primary care program developed in 98 patients with mild-moderate disease. In this program no patients needed hospital admission, but they were significantly younger and up to 40% had no comorbidity.^13^ Barroso et al. in an observational study of 122 patients with Covid infection from two cohorts in a primary care health center. In this study, covid infection predominated in patients without comorbidity, with a mean age of 52.7 years. 9.8% of patients required hospital admission and 1.6% died.^21^

Sitammagari et al*.* conducted a virtual follow-up study of patients with Covid infection. They included 1293 patients, of whom 40 (2.7%) required hospital admission and 2 patients died (0.14%). Patients were younger and had fewer comorbidities than those in our study, which facilitated the virtual follow-up that was carried out and may justify the lower percentage of hospital admissions.^17^

In our study only 7.2% required hospital admission. Considering that this is a population with comorbidity, the percentage of admissions is low. As expected, the risk of admission increased when the patient had two or more vulnerability factors. In this sense, Haddad et al., included in a remote patient monitoring program, patients with positive test and one or more risk factors for severe COVID-19 illness as defined by the US Centers for Disease Control and Prevention and expert consensus.^22^ Patients were compared with a control group, had a significantly lower rate of one or more hospitalization (13.7% vs 18.0%) and intensive care unit admission (2.3% vs 4.2%),^23^ which were higher than those described in our study. In addition, several vulnerability factors were associated with an increased risk of admission, such as diabetes mellitus, ischaemic heart disease and obesity, all of which have been widely described in the published literature.^24–28^

A noteworthy finding is that tachypnea and oxygen saturation were found to be predictors of admission and mortality. There are studies that describe whether the taking of vital signs by general practitioners is related to referral to the emergency department^29^ and several authors have showed in virtuals models of care with covid patients that some clinical clinical features measured at home are predictors for hospitalization.^30,31^ Identifying tachypnoea may promote faster response and earlier and more effective treatment options. Although there is ample evidence that altered respiratory rate is a predictor of adverse clinical events, it is a clinical sign that is often not recorded by healthcare workers.^32^ On the other hand, training patients and family members in respiratory rate recording could be useful in-home surveillance programs to detect respiratory deterioration early and activate emergency services. It is a clinical data that is easy to obtain, immediate, inexpensive and in periods of high incidence of respiratory infections could help to better identify the severity and optimize the use of resources.

The overall mortality rate was 0.7% (27 patients) and no patient died at home. Reported studies confirm the safety of home monitoring for suspected as well as established COVID-19 patients and demonstrated the potential of home monitoring to reduce hospital admissions by safely surveying clinical symptoms and vitals.^33^ However, we have not found any studies that refer to out-of-hospital mortality in patients monitored at home. Mortality rate is substantially lower than that described by other authors^34,35^ and similar to mortality described by Haddad et al*.*^23^ Also, predictors of mortality were respiratory rate > 22 breaths per minute, patients with three or more vulnerability factors, oxygen saturation ≤ 92%, chronic respiratory disease and active malignancy. These findings agree with those described in the literature.^36^ We have not found an explanation for such a low mortality rate in patients with vulnerability factors, but it is probably due to close monitoring of patients with early referral to the hospital.

This study has several limitations. The choice of patients was made by means of a computer program that evaluated the medical history and selected all patients who presented one or more of the vulnerability factors described, and these data were probably not up to date. Perhaps there were patients who had comorbidities and were not selected by the program. On the other hand, although approximately 49% had not received any vaccine dose, patients were included in the different stages of vaccination, which may influence the rate of hospitalization and mortality. Also, the retrospective design of the study does not allow the establishment of causal links between COVID and hospital admission or mortality. We acknowledge the limitation that we did not include a healthy control group, however, our purpose was to study the behavior of COVID-19 disease in vulnerable outpatients. The strengths of the study are the large sample size and the extensive collection of clinical variables in outpatients. Enhancing telemedicine can help to development new programs for primary care practice that extend beyond the current pandemic and improve the quality of health care.

## CONCLUSION

Patients with mild to moderate covid disease can be safely managed at home by a trained primary care team. The supervision of vulnerable patients by a Primary Care team was effective in the follow-up of these patients with complete resolution of symptoms in 91.7% of the cases without requiring hospital care. Training family members in taking vital signs is an effective strategy for identifying predictors of severity.

## Data Availability

The datasets during and/or analyzed during the current study available from the corresponding author on reasonable request.
